# Conflicting perceptions on participation between citizens and members of local government

**DOI:** 10.1007/s11135-017-0565-9

**Published:** 2017-09-04

**Authors:** Seyed Hamid Mohammadi, Sharifah Norazizan, Hedayat Allah Nikkhah

**Affiliations:** 1 Department of Social and Development Sciences, Faculty of Human Ecology, Putra University, Serdang, Malaysia; 2 Department of Social Science, Faculty of Human Sciences, Hormozgan University, 9 km road of Minab, Campus University of Hormozgan, Bandar Abbass, Hormozgan 79161-93145 Iran

**Keywords:** People-centered (PC) view, Authority-centered (AC) view, Citizen participation, Conflict perception, Local government

## Abstract

There is a growing consensus among citizens and members of local government that citizen participation is desirable for local government. However in practice, there are differing perceptions between them regarding the level and extent of citizen participation. As citizen participation is a multi faceted concept, its meaning is construed differently by both the members of local government and the citizen groups. This paper attempts to describe the conflicts that arise from participation. The paper is based on the study of the process of citizen participation in local government carried out in Torbat-Heydarieh city, Iran. A qualitative research method is seen as the most suitable approach of collecting and analyzing the data. The method employed for data collection in this research is in-depth interviews. In-depth interviews were conducted to gauge the existence of conflicts regarding participation. The findings reveal four areas that give rise to conflicts. This study also attempts to highlight two different views regarding citizen participation; the people-centered view and authority-centered view.

## Introduction

People participation is considered an important factor that contributes towards the success and prosperity of local government. According to Lowndes et al. (2001), people participation in local government produces better outcomes and promotes good governance. Although there is consensus among local governments and people that increased people’s participation is necessary and valuable at the local level, the former however are not really keen on people participation. According to Stivers (1990), the key question is the extent of citizens involvement in public decision making. Are citizens capable of making policies and decisions or should the members of local government make all decisions based on their knowledge, isolated from people’s opinion?

This subject has become important with the growth of citizen’s demand for participation in decision-making. It also has become more important with the creation of new regulations and laws by International Donor Agencies such as World Bank, IADP, and national governments to expend greater efforts to encourage citizens to participate in local government affairs. The issues on the role of citizen participation in local government matters have been highlighted by a number of researchers such as Mariana (2008) Kweit and Kweit (2007), and Roberts (2004). Mariana (2008) in her study argued that there is conflict between local authorities and people regarding peoples’ preferences and the extent of people’s participation. Lowndes et al. (2001) and Goss (1999), stated that the conflict between local government and people is focused on the difference in perceptions over participation. In Iran, the members of local government consider citizen participation as an advantage in fiscal terms rather than in terms of its administrative and intellectual potential. This is not what the people want (Ghanizadeh 1999).

This research has two key objectives. Firstly to recognize and distinguish two different viewpoints about citizen’s participation, one which is related to citizens’ perception of participation and another related to perception of members of local government. The second objective is to investigate the conflicts those arise over perception of public participation between the local government’s members and citizens. A better understanding of conflict perception is necessary if citizens and members of local government are to address the very real problems that exist between them. In this paper, for identification of the root causes of conflicting perceptions, the theory of conflict was helped. As Seymour (2003) points out, values such as power distribution and resource control contribute to formation of the perception. The conflict theory is connected to power relations.

## Literature review

Citizen participation refers to a range of different actions by different people (Pateman 1970). According to Day (1997) it is a contestable concept. Some community groups welcome citizen involvement in decision-making, but others do not. Wilfred et al. (1973) pointed out how people participation “has different meanings for different individuals and interest groups”. For some, it means the traditional elitist act as advisory functions in which only a limited group makes the decisions. For others, participation ensures that people work in the centre, voice local needs, and implement the programs. As Wilfred et al. (1973) argued, whenever a concept has multiple meanings, its application has potential for conflict. When a concept is interpreted by two or more interest groups, conflicts will surely occur. Roberts (2004) and Tomas (1999) stated the role of people participation in the decision-making process “has sparked much debate”.

In this study, by reviewing the literatures of citizen’s participation in local affairs, two different views about citizen’s participation is distinguished; the first one, advocate of citizen participation in decision-making processes. This view is close to citizen’s view, that confirms the participation of people in all aspects of local government activities, and, in this paper it is called People-centered (PC) view or direct participation in Robert’s term (2004). The second view is known as Authority-centered (AC). This view believed that the local authority has control over the people. Based on these two perspectives, it shows that there is a conflict between the people and the local authority. Both parties, the people (or citizens) and the local authorities (or local government members) are struggling to influence the decision in managing local affairs in one locality or municipality, for an example. The central issue is about power and the ability to exercise power over the other. The proponent of PC perspective views that it is the people has the rights to determine their lives in their own locality, and therefore it is the role and obligation for them to actively participate to decide for themselves. Influencing the decision making is a must and a critical agenda for them. Leaving the decision making process to the local authority is perceived as though their rights have been surrendered to other party, that signify they have lost their demanding and persuasion power.

Different arguments have been stated by different authors or researchers to advocate people participation. These arguments reinforce the role of people in the decision-making process. In his study, Roberts (2004) summarized some of the basic arguments in support of peoples’ participation. He expressed direct people participation as “developmental”, “educative”, “integrative”, “legitimating”, “instrumental” and “realistic”. According to Roberts (2004), participation is what enables people to be their own masters, and it ensures that no person or group is the master of the other. Despite that he also warned that direct participation is sometimes unrealistic and disruptive. In a similar vein, Salisbury (1975) believed when the opportunities have not been provided for all citizens to participate, and somebody prevent of participatory process, it causes a division between those who participate and those who do not. This ultimately produces conflicts between participants and non-participants.

Based on PC view, citizens become active participants in the creation and implementation of the policies, decisions, and process which affect them. Citizens are capable individuals who are willing and able to take responsibility for their own choices, decisions, and action although one may says this is not always the case, i.e. unrealistic (Roberts 2004). Roberts (2004), Box (1998), and Gaventa (2006) who support the PC view claimed that citizens are capable and efficient to participate in all aspects of local government matters. This is because people learned through participating and thus the educative element of participation (Roberts 2004) will make people more matured.

In contrast, the AC view, which is supported often by members of local government or elected officials, those who believe, direct citizen participation is politically “naive”, and governance should rest on informed and knowledgeable elite. Only a small group needs to be actively and directly involve in decision-making. Some scholars such as Yang (2006), Rosenbaum (1978), Bowman and Kearney (2007), Crosby et al. (1986), and Irvin and Stansbury (2004), argued that the local authorities focus on a limited scope or role for people’s participation in the decision-making process and the formulation of policies. According to Bowman and Kearney (2007), from the perspective of local government officials, people participation can be a nuisance because it may disrupt established routines.

Consistent with the perception of local government members on participation, a number of researchers have stated some arguments that prevent direct people participation. Fishkin (1991) and Stivers (1990) expressed how modern societies are too complex, and so it is difficult for government to support face-to face relationships. Cleveland (1975) believed mass participation is undesirable because it would be too expensive, too slow and a waste of time. Moreover, the majority of people do not have the capabilities to manage the complex issues facing modern societies (DeSario and Langton 1987; Fishkin 1991). This is because management needs skills, resources, money and time, which most people do not have (Grant 1994; King et al. 1998). In addition, people are too busy with their private lives, including supporting their families. All this issues prevents direct participation to take place.

According to Barber (1984), excessive participation by citizens increases political conflict; it jeopardizes stability and social order. High expectations for direct participation is difficult to meet, thus it creates alienation, low self-esteem, and distrust and this discourage participation of people (Kweit and Kweit 1981). In contrast, limited scope of citizen’s participation has advantages, as it undermines “the shock of disagreement, adjustment, and change” (Pateman 1970, p. 7).

Based on the literatures, it can be concluded that the most important element that differ PC from AC views is the level of people participation in the decision-making process. This, of course, is about power. So members of local government advocate AC in an attempt to keep power for themselves, and to avoid sharing their power with citizens. On this view, power is under local government control, and they are not willing to confer power to the people. But, on the other hand, with PC, people seek to be involved in the decision-making process and pursue the redistribution of power. That is, power to decide, to act, and to achieve what they aim for in an open system where bargaining is possible, influencing and adjusting to the needs and situations of the people is permissible. In such a realistic environment PC view of participation could allow empowerment to take places within the acceptable regulation, norms and practice. This is not easy and very challenging task for both party, i.e. the people and local authority. The reconciliation is based on how one sees power, power relation and power being exercised. If power is seen to be a reducible entity, then it’s creating the dichotomy polemic between the team that ‘have-power’ and the team that ‘do-not-have power’. On the other hand, if power is views as a facilitating mechanism to achieve certain common goals that can benefitted both party, then power is everywhere to be shared and negotiated among the interested parties.

**Fig. 1:**
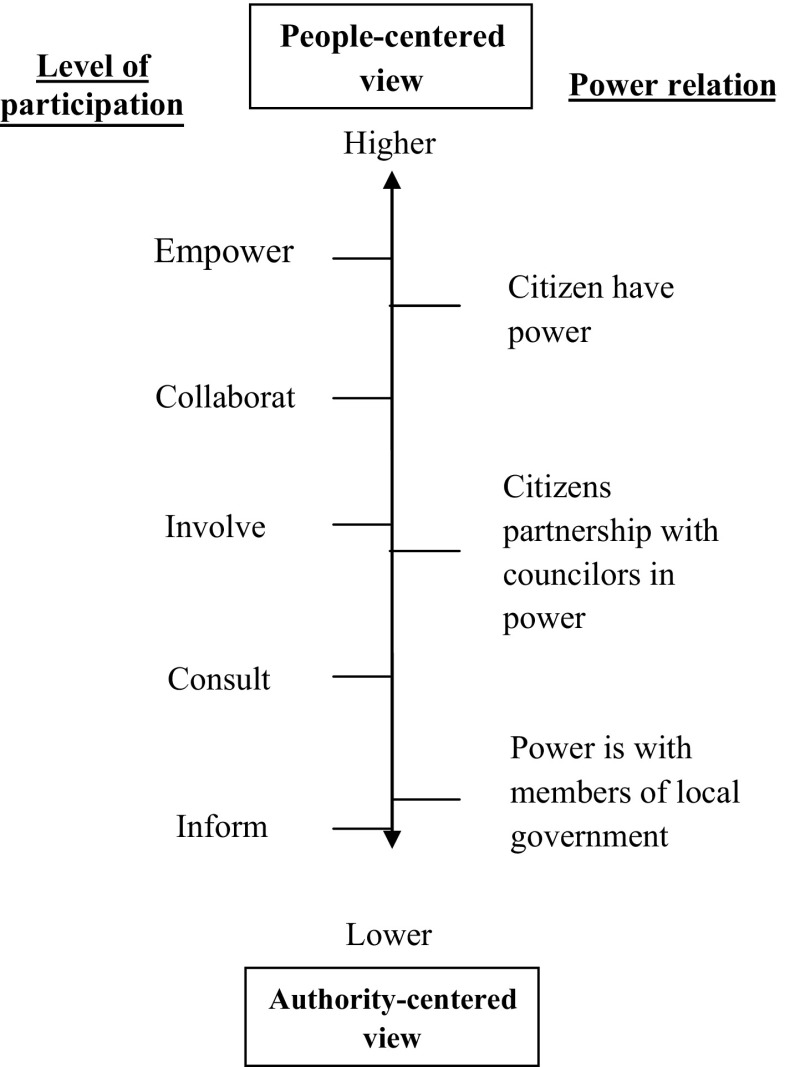
The citizen’s participation continuum. Adopted from Silverman (2005)

The continuum introduced in this article begins to identify the level of people participation that would be associated with various levels of power sharing and power relations between citizens and members of local government. This approach was used by Silverman (2005) to categorize formal societal organization in relation to participations tools. In this study, in the left side of Fig. 1, high levels of people participation such as decision making and has-delegated levels are associated with level of citizen power. Although the low level of participation such as manipulation and informing are associated with power is dominate by members of local government. In addition to predicting in which level of participation citizen partner with councilors in power, this framework predicts in consultation level, power is being divided between citizens and members of local government.

In his study, Yang (2006) noted in general local government views people as occasionally selfish with their focus limited to short-term personal gain rather than long-term community interest. Rosenbaum (1978, as cited in Yang 2006) mentioned there is a past viewpoint in which people are considered incompetent in public decision making. Bowman and Kearney (2007) mentioned that members of local government deemed people participation can disrupt established order. Incorporating people participation into ongoing operations is a strenuous challenge.

According to various issues highlighted by different authors, as well as whatever was described in the PC and AC views, it is clear that there are some differences in perception of participation between people and local government, and most of these differences are related to an extension of people’s participation and power relations as perceived by the two parties. According to Lowndes et al. (2001) and Goss (1999), local government members’ perceptions of people participation is often different to the peoples’ perception toward participation. When the literature on conflict of participation between members of local government and people is reviewed, the roots of conflict is valuable to understood and venture. Hence in this study, the theory of conflict was helped to explore how conflict perception arises between people and local government. Since conflict theory is connected to power relations, it helps towards a better understanding of the difference in perceptions of participation in the decision-making process.

### Conflict theory

According to conflict theory, the groups or individuals that have power, imposes their interest on the groups without power, in order to maintain their power position, as well as order within society (Delaney 2005, p. 70). The possession of power is critical element in conflict theory. Dahrendorf’s view support this argument that, power and authority does not reside in individuals but in positions. The obvious implication of this social reality is that conflict is inevitable (Delaney 2005, p. 71). The conflict perspective acknowledges that there are several groups fighting over scarce resources of society. Each group attempts to have power and control over others. Conflict theorists believe that power is the core of all social resources. Thus, conflict theory views society as composed of competing elements that fight over scarce resources, especially on power.

**Fig. 2:**
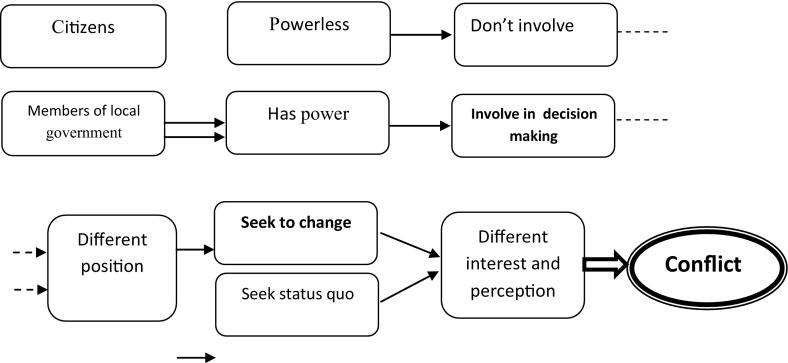
Conflict perception between citizens and members of local government

The councilors who are in dominant position seek to maintain their preponderance, while citizens who are in subordinate positions seek to change and they do not believe to the interest of councilors therefore, conflict of interests occurs between local government members and citizens. Actually, member of local governments that are in power seek to maintain the status quo, while citizens without power are attempt to struggle for some level of power and control. It will be mentioned further, the meaning of power in this study is control over decision-making process. So, those who have power are involved in the decision-making process. As shown in Fig. 2, this theory believed that there is a struggling of power between two parties, which lead to different level of treatment give and receive by the parties involved, i.e. between the powerless and the power holder, and eventually prescribed the type and degree of participation between the two. In this uneven power distribution, the power relation and exercising of power by individuals (members of local government) who are in power could strengthen their dominance over the powerless. Due to the parallel mode of relation (as shown in the figure), the divergence of presumption between the power holder and the people and the different interest and perception among the parties involved, consensus is difficult to achieve, instead conflict continue to sharpen.

## Methodology

This study was qualitatively approached. Qualitative research allows the researcher to draw upon a variety of research methods to probe the relationship of theory, philosophy, and objectives to practice by examining complex societal or public issues in depth and detail. (Guba and Lincon 1985; Maxwell 1996). This method is particularly suited to studying issues, relationships, organizations, problems, and other anomalies (Mohr 1982). “The strength of qualitative research derives primarily from its inductive approach, its focus on specific situations or people, and its emphasis on words rather than numbers” (Maxwell 1996, p. 17). Qualitative research methods nowadays are widely used in participation research and are gaining wide acceptance in the social sciences (Kirby and McKenna 1989; Maguire 1987; Mies 1983). In participation research, anthropologists and sociologists have used qualitative research (Oakley 1991).

In-depth interview technique was used to collect the data. The technique is most often used in public administration research (Berg 2001). Qualitative interview allow the flexibility and rich detail necessary to address an issue as complex as citizen participation. Maguire (1987) stated the importance of in-depth interview for assessing the participation. This type of interviewing technique allows researcher, going to detail of topics, formulate deeply questions, and move in new directions (Gubrium and Holstein 2002, p. 57). Howden and Vanclay (2000) argued interview is a useful method for local community research. The perspective and experience of respondents are used to describe the process of decision making in local government.

The findings of this paper are drawn from 40 interview carried out with citizens of Torbat-Heydarieh. Torbat Heydarieh is located in the east north of Iran in Khorasan Razavi province; it is 1005 km far from Tehran (capital of Iran). Particular attention was paid to recruiting citizens from various sectors. There were four different types of citizens as respondents. Interviewees were as follow;

- Community leaders or representatives of local people (n = 11). According to Thompson et al. (2000) community leaders are appropriate respondents for assessing community affairs.
- Members of local government (n = 7), who are individuals involved in the decision-making process.
- Members of advisory board/committees (n = 7) who participate in local government initiatives. According to Mariana (2008), individual who attend in local government are good key informants for interview about local government processes.
- The heads/representatives of private sectors, NGOs, and university lecturers (n = 15) who were chosen because they represented the voice of the specific group of people (Eng and Parker 1994). These are citizens who expected to be involved in local government by expressing their opinion and idea. Therefore, interview concentrated upon individuals who had some knowledge of, or contact with local government. In this study, the community groups consist of; community leaders, advisory bodies, and representatives of public and private sectors, whereas the members of local government are considered as the power-holders. In the data collection step, the researcher investigate the manners of decision-making, participatory process, and forms that people be involved in local government. In processing and analyzing the data, this study followed of procedure that has been described by LeCompte and Schensul (1999) with adjustment.

### Qualitative interview

In this research, a total of 40 individuals, comprising community leaders (n = 11), advisory boards (n = 7), NGOs (n = 4), private sectors (n = 4), lecturers and university researchers (n = 3), former members of local government (n = 4), and members of local government (n = 7) were interviewed. Their opinions and perceptions regarding the problems of participation and decision making process in Torbat local government were sought through these interviews. This was conducted using a semi-structured questionnaire and in-depth interviews. The respondents were participants in the local government process, members of the main committee, community representatives, and other various working groups.

The interview questions were designed to capture some views and inputs with regard to the people’s participation. The respondents were asked about their feelings, experiences, attitudes and expectations of participation in local government. Their understanding of the participation in local government, and the reasons for participating were assessed. Their motives and expectations were also probed. Their opinions about those who are invited or involved in local government sessions were asked. Finally, they were requested to express their future expectations of local government plans. Although the main interview guides were targeted at participants in local government matters, separate interview guidelines were designed for different groups of respondents.

### Data analysis

Qualitative data analysis is the range of processes and procedures whereby the qualitative data collected is transformed into some form of explanation, understanding or interpretation of the people and situations investigated. Qualitative data is often subjective and rich, consisting of in-depth information normally presented in the form of words. Analyzing qualitative data entails reading large amount of transcripts, looking for similarities or differences, and subsequently finding themes and developing categories. Collected qualitative data will sort and code thematically into useful themes. The codes emerged from the data as well as from a priori established ideas in the literature and the findings of the quantitative data (Stroh 2000). Such a process allows comparing and contrasting ideas in the interviews, and checking the substance of the codes by constantly referring back to the original interview texts (Stroh 2000).

In processing and analyzing the data, this study followed the procedure described by LeCompte and Schensul (1999) with some minor adjustments. The collected data (by interviews) was organized for the purpose of analysis. Analysis involved ‘tidying up’ data, organizing them in files, labeling the files, as well as organizing and reducing the data according to the ideas, themes, units, patterns, and structures within them that are beginning to become apparent. This involves some form of coding or categorizing data (LeCompte and Schensul 1999).

### Reliability and validity

In order to establish the trustworthiness of this study and to validate the findings and subsequent conclusions, the following strategies were adopted.

According to Merriam (2002), multiple sources of data is the most well-known strategy for shoring up the trustworthiness of a qualitative study. Merriam (2002), emphasized in particular the common use of multiple data collection methods in qualitative studies. In this study therefore, the researcher employed multiple methods as a strategy to increase the rigor, legitimacy, and trustworthiness of the study. In this study the researcher collected data through a triangulation approach which utilized survey questionnaire, in-depth interviews, and documentary sources.

Triangulation is typically a strategy (test) for improving the validity and reliability of research or evaluation of findings.

In a multi-method study, the researcher has the opportunity to improve validity and reliability of data. Document sources were also sought to establish reliability and validity, such as the minutes of meetings of local government, local government documents, and local government documents.

In addition, the researcher used an audit trail. Guba and Lincon (1985) said that an audit trail is established to demonstrate trustworthiness. According to them, it enables an external reviewer to form opinions about the products of the study. An audit trail is also used to describe the process of data collection, the construction of categories, and “how discussions were made throughout the inquiry” (Merriam 1998, p. 172).

## Results and discussion

The second objective of the study is to investigate the conflicts arised over the perception of participation between the members of the local government and the people. This study used in-depth interview to explore this objective. As was mentioned, there is a difference in perception between people and members of local government about people’s participation. Lowndes et al. (2001) and Goss (1999), argued that the perception of members of local government about people participation often is different from citizen’s perception toward participation. There are four important issues identified with regards to the perception towards participation held between the members of local government and the people.

The first issue indicated by the informants was related to power sharing and decision-making by the members of local government. The local government was established in cities to involve people in their affairs. People must participate in decision-making and planning but that was not implemented. The members of the local government are not willing to share power in decision-making with other stakeholders. The elements of good governance which include transparency, being open, sharing of power and consensus building were absent and that is a source of conflicts leading towards negative perception towards participation.

The second issue contributed towards conflicts on perceptions towards participation is related to the extent to which people participate. There are different views between members of local government and the local people regarding the extent of participation. The members of local government are ambivalent about people’s involvement in decision-making process. In contrast, the people are willing to participate in planning and decision-making process. The people argued that they have the ability to be involved in high level of participation. The people argued that the members of the local government to be more efficient in performing their roles and functions.

The third issue regarding conflict of perceptions towards participation between members of local government and the local people concerns the failure on the part of members of local government for not inviting the local people as experts in plan making. Policies formulated and implemented do not involve the local people and purely adopting top–down approach. The local people argued that long term programs should be planned and developed involving the locals during its inception right through its implementation. With regard to making decisions, the respondents argued that decisions are made based purely on the members of local governments’ views and opinions. These views and opinions may sometime contradict with local people. According to them, the members of local government need to understand that the desire to make changes and to influence decision-making process is one of main reasons why people participate in local government issues.

The forth issue that indicates to conflict perception is about discrepancy of point of view. Regarding to this issue, there is no common definition of participation between people and members of local government. Most probably, the members of local government limit the definition of participation only to fiscal participation, while people define participation in all its aspects. Also, according to the members of local government participation means that, following and accepting the policies and decisions that are taken by local government, whilst form the peoples’ view, participation means that to play an active role in decision making process.

As mentioned earlier, one of the main objectives of this study is to investigate conflicts arising from perceptions of public participation between the local government members and citizens. The in-depth interviews revealed the critical importance of four areas that cause conflicts namely, power sharing and power relations, the extent of people’s participation, planning and decision-making process, and discrepancies in the views about people participation.

### Power sharing and power relations

One of the main issues highlighted by the informants is related to power sharing and decision-making by the councilors. AB6, CR2, L1 and FC4 commented that local government were established in cities to elicit people participation . People should participate in decision-making and planning, but there are some problems, which are related to councilors, FC4 argued that;

the councilors are not willing to involve the people in higher levels of participation, … our councilors have to change their mind. They must not feel that they can do everything themselves. So, the key principle is the councilors must change their views and mind towards peoples’ participation.

This is closely related to the element of power sharing in the process of decision making with other stakeholders. According to L1:

The members of the local government are not willing to share power in decision-making with other stakeholders and want to hold the power to make decisions to themselves. The element of transparency, sharing of power and consensus decisions could be the reasons for conflicts among the community and members of local government.

CR9, L2, FC3, and AB4 commented that members of the local government must understand that policy-making and the process of decision-making are among the reasons why people wish to participate in local government.

The respondents, representing the community representatives (CR3, CR10), expressed their concern about the lack of transparency and power sharing with the councilors. According to them community representatives did not trust members of local government. Most of them felt consensus and transparency in decision-making were lacking in local government. They felt that the decision-making powers were not shared with them as part of the community. The majority of the stakeholders were also dissatisfied with the decision-making process (CR3, CR4, CR11, AB3, AB4, FC3, NGO1, PS2 and L1). According to CR6;

….. the councilors made decisions without taking into account the people’s opinion. If the decisions taken had considered the majority of the local people’s ideas, then there would be lesser conflicts/opposition and the implementation of policies would be easier.

NGO1 stated,

once selected, the members of the local government feel that they do not have to work with the people and they could manage/handle all issues and design policies by themselves.. They pay attention more to the government’s than the people’s needs. … the local government try to follow governmental policies instead of fulfilling the local people’s wishes.

On the other hand, people feel that local government take whatever actions they want without seeking the responses of the people. It means that people’s idea and demands are not taken into account by councilors (NGO3 and PS3).

A number of informants from community groups (except members of the local government) pointed out that they expect to be consulted before decision-making by councilors, they are interested to be involved, give suggestions and have opportunities to participate in affairs related to them and which affect their districts. They strongly expressed their dissatisfaction and their distrust towards management by the councilors and the current decision making process (CR3, CR4, CR9, AB3, AB4, FC3, NGO2, NGO1, PS2 and L1).

NG2 and CR11 alluded to the role of influential people in the process of decision-making and planning in local government. According to CR11 “influential persons affect the process of decision-making in local government and, since these people just pursue their own interests, they impose their views on local government without taking into consideration the views of the majority of the people.”. The respondents from advisory boards, private sectors, NGOs and former members of local government pointed out that community leaders/representatives should be involved actively as mediators in assisting the councils to explain participatory issues and local government targets to the people . Trust is necessary to be created between councilors and community representatives, and local government must be open and accept the comments of community groups in order to create this trust (L1, L3, FC2, NGO2, PS2 and PS4).

### The extent of peoples’ participation

There are different views between councilors and the locals concerning the extent of involvement of the people in L.C issues. One of the academics (L2) pointed out that generally participation is divided into two types; general participation and planned participation. According to L2.

general participation would not create efficiency except in certain circumstances . In this type of participation, people do not take into account the decision-making process as it is “top-down” and is imposed on the people without public involvement.

However planned participation is demand-based not supply-based, and decision making is with the people, not for the people. Demand-based participation is to inform people, on the things that needs to be done and seek peoples’ opinions and views about the programs and implementation of the plans. In this type of participation, people are involved in policy-making, decision-making, implementation and evaluation. Actually the extent of this participation is desirable for people.

FC1, PS1 and PS3 pointed out that councilors only consider fiscal participation, whereas notional/mental participation must be the first step, as it is more valuable. L.C must consider a wide range of views to improve its efficiency. Actually it is a principle that has been accepted by most theorists. Many of the community groups stated that people have the ability to be involved in higher levels of participation. (?) (CR2, CR5, L3, L4, AB2, AB3, and FC2). NGO3 added that “local government is a governmental and public institution . Therefore the entire decision making must be by the people”. (oriented from people’s ideas).

According to PS2;

Local government must involve the people in local issues because they are more aware of the local problems. People are keen to take part at the implementation, monitoring and decision-making levels, but councilors are not interested, because they are afraid of losing their authority and power especially when there have been abuses from their position in local government.

When enquired about the extent of public involvement in local government issues, CR3, CR8, AB1, AB5, L2, FC2, FC3, NG1, PS2, MLC1, MLC6 pointed out that irrespective of the rate of people participation, the efficiency of the local government will be improved. It will also garner people support and acceptance of decisions and plans made by the local government.

As mentioned in chapter 2, although there is theoretical and practical recognition that the public must be more involved in decision-making, many councilors are at best, ambivalent about public involvement, at worst, they find it problematic. They argue that people participation delays decision-making and implementation processes.

MLC1 expressed that;

at the conceptual level there are no differences in opinions between the locals and members of the local government. Both groups know that participation is useful and improves the quality of local government administration, but in practice there are some differences.

### Planning and decision-making process

According to the Iran’s constitution, local governments are established to expedite the implementation of social, cultural, and welfare programs with the cooperation of the people. According to the laws, local governments could establish various special commissions and invite locals, experienced people and experts as advisors to undertake programs and resolve urban problems. Despite being mentioned in the constitution, a number of informants (CR7, CR8, L1, NGO2, NGO3 and PS3) pointed out that local government do not invite and utilize locals and experts for designing their plans. Policies are taken by members of the local government and imposed on the people. This was purely a top–down approach adopted by the government.

A number of NGO groups and community leaders mentioned that local governments do not have long-term programs to increase their efficiency and most of the sessions are held mainly to resolve daily issues and respond to people’s demands (NGO1, NGO3, CR1 and CR9). Local government should pay attention to macro issues and long-term plans, rather than on micro and primary issues. AB2 expressed that,

the local government of Torbat is not plan-based. Their sessions are not for designing plans and deciding on the approaches to get the plans. Their sessions are just for resolving minor problems and superficial affairs. According to the law, the long-term programs for the city should be designed by the local government.

According to CR2, “the majority of the councilors do not have the knowledge and capability to examine the social, cultural and welfare issues and limitations of the city, due to their lack of expertise ”.

Furthermore, NGO1 stressed, “councilors are unable to prepare reformative programs. Most councilors are not familiar with arrangements for programs and city services systems and to attract people participation”.

Based on interviews, 17 informants, not including the councilors, pointed out that the plans of the local government are designed by its members, 6 informants stated by specialized commissions of local government, and the rest (2 informants) stated that local governments have no plans at all, so, it is meaningless to say, how local government prepare their plans.

A majority of the respondents said that the local government programs do not represent the views of the community (CR3, CR5, CR7, CR11, L2, L3, FC2, and PS2). One of the community representatives (CR8) said that “if local government’s plans reflect the community’s views, the rate of people’s dissatisfaction will be lower than seen in present days”.

With regard to how decisions are made in the local governments, most of those interviewed (CR5, CR7, CR10, AB2, AB7, L2, L3, FC1, FC4, NG2, NG3, PS3, OS4, MLC2, MLC3, MLC6) pointed out that the decisions are made by the members of local government based on their own views, which in fact are moulded by their own experiences and knowledge. CR11 and NGO2 also said that influential persons and governmental authorities/people effect and play a role in the decision-making process.

CR3, CR9, FC2, L1, and AB6 were of the opinion that urban issues and problems are complex and are determined only by members of the local government. Top–down decision making would not resolve the urban problems and issues. L2 argued;

It is necessary for local governments to share power with their stakeholders. Through this the stakeholders are able participate in local government issues voluntarily and regain their role as active people . The members of the local government have to understand that the desire to make changes and influence decision-making process is one of main reasons why people participate in local government in the first place.

As backbone of the city, people involved in the decision-making process have a sense of responsibility towards their city. This facilitates speedy resolution of problems faced by the local government. (AB1).

In this study, 28 informants stated that they do not agree with this process of decision-making. According to PS2 “… because the councilors are not experts in various fields, their decisions are not based on knowledge and expertise, rather decisions are based on personal benefits”. On the other hand, CR2, AB5, AB6, NG4, PS agree that councilors have to make decisions unless before any decision-making, opinions of experts and advisors are sought . AB6 argued that “this type of decision-making speeds up the process while a high – level committee should be held responsible for these decisions”.

### Differences in viewpoints about people participation

Based on interviews with key informants, it can be concluded that there are some differences in the viewpoints between the councilors and the local people with regards to the concept of participation which lead to conflicts. CR1, CR5, CR9, CR10, AB4, AB7, L2, L3, and NGO3 pointed out that the definition of people participation to understand by the local government completely differs from that of the local people.

AB4 stated that “the members of the local government prefer fiscal participation as they want people to pay their taxes and tolls, which they consider as adequate participation”. One of the members of the private sector (PS1) expressed,

The most important concern of councilors is payment of salary to their staffs and to themselves. So, they emphazise fiscal participation, because they can earn income through this but not through participation in decision-making and monitoring. This definitely is in contrast to people’s approach towards participation, as people wish to participate at the decision-making, evaluation and implementation levels.

NGO3 confirmed this opinion and added, “the councilors pretend to welcome people and they present unrealistic statistics about people participation in social activities of the local government”. 264.

One of academics (L1) stated that:

… people expect their demands to be considered and supported by the councilors. On the other hand, councilors expect the people to follow the decisions and plans designed by the local government. Since the views and opinions of people don’t integrate with plans and decisions of the local government, they are in conflict with each other. … The approach of the local government towards people participation is an instrumental approach, and members of the local government look to people participation just as tokenism.

When asked about the reasons for differences in the viewpoints, the majority of the community groups indicated two factors: Firstly, power relations, and secondly, councilors are themselves more skilled. CR8, CR10, L2, L4, NGO1, AB6 and AB7 mentioned that due to their positions, councilors have some legitimacy and power, which they do not wish to devolve to the people. They therefore prevent people’s involvement in decision-making. CR9 stated that.

councilors have some authority and they oppose those who want to limit their power. One of these limitations is people’s monitoring, which arises from their active participation.

An NGO member (NGO1) said that there is a proverb in Iran “great men attract great people”. Hence when members of local government are not experts they don’t accept people with expertise for consultation, because they feel, they would lose their power and positions.

According to MLC5 and AB2, there are no differences in the perceptions concerning concept of participation between local people and M.LC. Both groups believe participation is useful, it improves decisions, increases mutual trust, and creates transparency. However they differ in the areas and extent of participation. MLC5 argued;

People must adjust/adopt themselves to the policies of the local government, as they are oriented towards improving their welfare and convenience. … the most important type of participation as far as the local government is concerned is people’s fiscal participation …. for the councilors it is desirable if people pay their taxes and tolls to the municipal council on time.

Another member of the local government (MLC2) stated :

As the people do not have enough knowledge and awareness regarding the need for involvement in the policies of the local government, it is better that the people just follow the programs designed by the local government. However the people could give their suggestions and express their opinions, which could either be rejected or accepted by members of the local government. … ordinary people are not willing to participate and there is little public enthusiasm for enhancing participation.

## Conclusion and discussion

Local government is a platform that all people can participate in local issues effectively. People participation is considered as an important factor for the success and prosperity of local government. However the role of people participation in local government matter is a debatable issue. Although there is consensus among local government and the people that people’s participation is necessary and valuable at local level, but most of the members of local government do not actively seek people ’ involvement. As mentioned earlier, there are two different views regarding people participation in local government. From the people ’ point of view, people should participate in all aspects of local government activities. Another view which is from the members of local government viewed that there should be a limit to participation of people in local issues, especially in decision-making process. Mariana (2008) and Kweit and Kweit (2007) in their study described a fundamental conflict between these views. On one hand, people are interested in participating in local issues that affect their lives, and on the other hand, the members of local government are reluctant to involve the people in the decision making process.

With regards to these tensions, it is interesting to note that in Iran there is a conflict of perception between the people and members of local government regarding people’ preferences, and the broad and intensity of people’s participation. Based on the in-depth interview the conflicting perceptions can be categorized into four themes. The four themes are power sharing, extension of participation, consensus on decision-making, and essence of people participation. A better understanding of the people’ perception towards participation is essential in providing the right situation for the people to participate in local government (Lowndes et al. 2001).

It should be noted that the enthusiasm or reluctance of people to participate in local government is strongly dependent on their perception towards participation. Hence, this prompt the need to investigate the different perception towards perceptions by the people and the councilors.

The first issue indicated by the informants was related to power sharing and decision-making by the members of local government. The local government was established in cities to involve people in their affairs. People must participate in decision-making and planning but that was not implemented. The members of the local government are not willing to share power in decision-making with other stakeholders. The elements of good governance which include transparency, being open, sharing of power and consensus building were absent and that is a source of conflicts leading towards negative perception towards participation.

The second issue contributed towards conflicts on perceptions towards participation is related to the extent to which people participate. There are different views between members of local government and the local people regarding the extent of participation. The members of local government are ambivalent about people’s involvement in decision-making process. In contrast, the people are willing to participate in planning and decision-making process. The people argued that they have the ability to be involved in high level of participation. The people argued that the members of the local government to be more efficient in performing their roles and functions.

The third issue regarding conflict of perceptions towards participation between members of local government and the local people concerns the failure on the part of members of local government for not inviting the local people as experts in plan making. Policies formulated and implemented do not involve the local people and purely adopting top–down approach. The local people argued that long term programs should be planned and developed involving the locals during its inception right through its implementation. With regard to making decisions, the respondents argued that decisions are made based purely on the members of local governments’ views and opinions. These views and opinions may sometime contradict with local people. According to them, the members of local government need to understand that the desire to make changes and to influence decision-making process is one of main reasons why people participate in local government issues.

The forth issue that indicates to conflict perception is about discrepancy of point of view. Regarding to this issue, there is no common definition of participation between people and members of local government. Most probably, the members of local government limit the definition of participation only to fiscal participation, while people define participation in all its aspects. Also, according to the members of local government participation means that, following and accepting the policies and decisions that are taken by local government, whilst form the peoples’ view, participation means that to play an active role in decision making process.

Good governance is driven by enhanced people participation and efficiency and accountability of local government. Since, people participation in local issues is the heart of local governance, it is thus the main factor in local development, with both at the national and local governments taking the lead.

Iran like every other developing countries, the dominance of government in policy and decision-making processes has contributed to the unfavorable environment for people’s participation. Nonetheless with increased demands for people participation in government activities such as participation in decision-making processes, the central government has established local government to encourage people participation at local level. The members of local government in Iran are attempting to bring a wide range of people for discussion and consultation, and established strategies to facilitate people’s participation. However, this is not on a full partnership basis and strategies are based on one-way relations. Local government has the potential to foster enhanced civic engagement and the research has found examples where this is occurring on a localized scale. However, the responsibilities of the local government has not been clear to the people, and that the function of local government as an avenue for the local people to be involved in the local issues has not been made known to the people. Also local government has not been successful in collaborative process to enable the wider potential of citizenship, and its potentials for the most parts are still underdeveloped.

In particular, this paper argued that conflict perception among stakeholders arises from power relation between them. Since, the position of citizens and local government members in community is different, then, their perception towards participation also differs. Meanwhile, it is difficult to have common perception on participation between local government and citizens. Therefore, the researcher highlights the conflict perception towards participation between them. This would enhance the understanding of participation between members of local government and citizens. A better understanding of conflict perception is necessary if stakeholders are to address the very real problems that exist between them.
